# Dynamically Changing Mental Stress Parameters of First-Year Medical Students over the Three-Year Course of the COVID-19 Pandemic: A Repeated Cross-Sectional Study

**DOI:** 10.3390/healthcare11111558

**Published:** 2023-05-26

**Authors:** Morris Gellisch, Martin Bablok, Gabriela Morosan-Puopolo, Thorsten Schäfer, Beate Brand-Saberi

**Affiliations:** 1 Department of Anatomy and Molecular Embryology, Institute of Anatomy, Medical Faculty, Ruhr University Bochum, 44801 Bochum, Germany; martin.bablok@rub.de (M.B.); gabriela.morosan-puopolo@rub.de (G.M.-P.); beate.brand-saberi@rub.de (B.B.-S.); 2 Center for Medical Education, Ruhr-University Bochum, 44801 Bochum, Germany; thorsten.schaefer@ruhr-uni-bochum.de

**Keywords:** stress, mental health, education, COVID-19, risk management, vulnerable populations

## Abstract

Numerous research results have already pointed towards the negative influence of increased mental stress on educational processes and motivational criteria. It has also been shown that the global public health crisis induced by COVID-19 was related to anxiety symptoms and elevated levels of distress. To holistically elucidate the dynamics of the pandemic-related mental stress of first-year medical students, the associated parameters of three different cohorts were measured at the beginning of the pandemic-related restrictions on university life in Germany (20/21), at the peak of the COVID-19-related restrictions (21/22) and during the easing of the restrictions in the winter term 22/23. In a repeated cross-sectional study design, the constructs of worries, tension, demands and joy were collected from first-year medical students (*n* = 578) using the Perceived Stress Questionnaire. The results demonstrate significantly increased values of the constructs worries (*p* < 0.001), tension (*p* < 0.001) and demands (*p* < 0.001) at the peak of the pandemic related restrictions compared to the previous and following year as well as significantly decreasing values of general joy of life during the observed period of 3 years (all *p*-values < 0.001). A confirmatory factor analysis was performed to verify the questionnaire’s factor structure regarding the addressed target group during the pandemic (CFI: 0.908, RMSEA: 0.071, SRMR: 0.052). These data, collected over a period of three years, provide information regarding dynamically manifesting mental stress during the COVID-19 pandemic, and refer to new areas of responsibility for the faculties to adequately counteract future crisis situations.

## 1. Introduction

At the turn of the year 2019/2020, the WHO Country Office in China received information regarding increased incidents of a novel viral pneumonia in the city of Wuhan, which, in retrospect, was the first signal of a developing pandemic [1]. In addition to global financial losses and drastic restrictions on leisure activities, 6.9 million reported deaths from COVID-19 infections occurred by mid-2022 (Institute for Health Metrics and Evaluation) [2]. The long-COVID syndrome appeared as an additional burdensome phenomenon, which describes long-term consequences of the virus infection, which can manifest themselves neurologically, psychologically and physiologically [3,4,5]. Emerging economic difficulties, the unpredictable nature of the COVID-19 pandemic and its consequences increase the risk of developing mental health problems, such as symptoms of depression and anxiety [6].

These serious consequences of the COVID-19 pandemic as well as the necessary regulations to limit in-person contact also posed an enormous challenge for educational processes in the relevant institutions [7,8,9,10]. In addition to self-reported declines in mental health and motivational factors, more objective changes such as different cortisol concentrations and altered sympathetic and parasympathetic activation patterns during remote learning compared to face-to-face learning could be demonstrated among German first-year medical students [11,12]. Mental stress among students, diffuse anxiety and difficulties in maintaining concentration during digital courses are widely reported burdens during the ongoing COVID-19 pandemic [13,14,15,16,17].

The extent to which these declines in mental health affect students’ learning processes can be discussed against the background of numerous research findings that have demonstrated a negative connection between depressive symptoms and academic performance, an effect that has been observed internationally and across different disciplines [18,19,20,21]. These findings could also be validated from the opposite direction by empirically demonstrating that students with higher mental health status show increased motivation in terms of academic achievement and learning success [22]. The underlying mechanisms rely on the detrimental effect of mental health symptoms on certain predictors of academic achievement, such as academic self-efficacy, persistence, and study skills [23]. These mechanisms are again of substantial importance to the particularly vulnerable group of medical students, since numerous studies have indicated that medical students have an increased risk of suffering from depressive symptoms compared to the general population [24,25,26]. These findings could be further substantiated by investigating a cohort of Portuguese medical students, who showed a significantly higher expression of anxiety symptoms compared to non-medical students [27]. It is imperative to consider these findings in the light of the theoretical concept that psychological capital such as hope, optimism and resilience are currently discussed as the main predictors of academic achievement as well as general well-being [28,29,30,31].

This insight becomes exceptionally critical in the context of crisis situations which—as in the case of the COVID-19 pandemic—reduce resilience factors due to social isolation, effectuating dramatic consequences for mental and physical health [32,33,34,35,36,37].

The emergence of the relevant construct stress follows the psychological concept described by Lazarus in 1966, which states that stress arises when the demands exceed the individual resources [38]. The effects of stress on cognitive processes must be considered in a differentiated manner, since a moderate arousal can exert positive effects on learning and memory processes, while persistent, chronic, and intense stress stimuli can cause both cognitive and health impairments [39,40,41,42].

While several studies have already examined subjectively perceived stress in medical students during the COVID-19 pandemic [43,44,45], there is a lack of repeated data collection comparing the perceived stress parameters at the beginning of the pandemic-related consequences on university teaching (20/21), at the peak of the pandemic-related consequences (21/22) and during the easing of the restrictions associated with COVID-19 (22/23), in order to shed light on the dynamic effects within the course of this crisis situation. This described comparison of three different cohorts of first-semester medical students during the different stages of the COVID-19 pandemic is the focus of the present study.

The basis of this study focuses on the question of the direct effects of the intensity of crisis situations on perceived stress of the particularly vulnerable population of first-year medical students. This study design is specifically aimed at illuminating study entry conditions of medical students in consideration of how the COVID-19 pandemic dynamics modulate stress-associated parameters. In addition, this project aims to validate the factor structure of the PSQ as a survey tool during an ongoing crisis situation.

## 2. Materials and Methods

### 2.1. Study Design

The interventions associated with COVID-19 to limit the risk of infection also had a considerably strong effect on the modalities of medical teaching in Germany. While the winter semester 20/21 at the Ruhr-University Bochum began—in parts—with regular face-to-face courses, the winter semester 21/22 started with a complete hybrid teaching design with a majority of distance teaching to consider the safety measures decided in Germany to reduce the COVID-19 infection risk. After this peak of the restrictions on in-person contact, the measures were eased at the beginning of the winter semester 22/23 so that regular face-to-face classes could be resumed. To make use of a survey instrument that also includes a resilience factor (general joy of life) in addition to stress-associated scales, the Perceived Stress Questionnaire (PSQ) was consistently used in this study for data collection for three consecutive years during the ongoing COVID-19 pandemic [46,47]. Levenstein et al. 1993 initially developed the PSQ for clinical psychosomatic research with a focus on the prognostic ability regarding the development of stress-related disorders [46]. The questionnaire consists of three dimensions of the stress reaction and one dimension of a stressor, which were deliberately chosen generically, enabling clinical utilizations of the PSQ as well as assessments in healthy adults [47]. In principle, the PSQ was identified as a valid and comprehensive assessment tool for stress research [48,49], which has already been utilized for the evaluation of perceived stress levels of medical students [26]. The 20-item instrument, which is subdivided into the subscales worries, tension, demands and joy, was used to assess the influence of the intensity of the COVID-19 pandemic-related consequences on perceived life stress. Therefore, the above-mentioned questionnaire was made available for first-semester medical students at the beginning of the respective winter semesters 20/21, 21/22 and 22/23 for three consecutive years. The questionnaire was completed at the university in paper format.

To investigate the academic performance of the students, the results of the final exam Anatomy I were used, since this exam provides an appropriate comparison due to its standardized form. In addition to the years 2020, 2021 and 2022, the academic performance in 2019 was indicated as a reference in order to provide additional information regarding the academic performance before the outbreak of the COVID-19 pandemic. Here, it should be noted that comparability is only possible to a limited extent, as the final examinations in the first semester at the Faculty of Medicine of the Ruhr University Bochum in 2020 were conducted online with only limited examination supervision, which was accompanied by an unusually high pass rate. While a uniform online exam policy is recommended for medical teaching, in which one camera should record the screen of the respective student and another camera the room [50], these technical configurations are often limited, so that the exam supervision in the case described here was limited to one camera, showing the students from the front during the online exam.

However, the pass rates for the years 2021 and 2022 can be used as a reference since these were conducted—as usual—in the presence of regular supervision.

### 2.2. Inclusion Criteria

All participants had to be properly enrolled medical students at the Ruhr University Bochum at the time of data collection. The recruitment for the study described here was deliberately aimed at including all genders, students of all ages as well as students from an immigrant background.

### 2.3. Participants

Five hundred and seventy-eight properly enrolled first-semester medical students (177 males: mean age = 21.52 ± 3.34 years; 399 females: mean age = 20.33 ± 2.90 years; 2 diverse: mean age = 19.50 ± 0.50 years (mean ± SD)) were participants in this study (Table 1). The observed COVID-19 restrictions explain the lower sample size in the winter semesters 20/21 (*n* = 126) and 2021/2022 (*n* = 116) compared to the winter semester 22/23 (*n* = 336).

Recruitment, data collection as well as obtaining informed written consent took place at the Medical Faculty of the Ruhr-University Bochum, Germany. The study procedure was conducted in agreement with the Declaration of Helsinki and approved by the ethics committee of the Medical Faculty at the Ruhr University Bochum (20–7135) and the ethics committee of the Professional School of Education (EPSE-2022-005).

### 2.4. Statistical Analysis

All statistical calculations were performed using R-statistical software. Factor descriptions were calculated, reporting the factor ratings, the mean value of each item of the factor, the standard deviation and the skewness (Table 2).

To validate the factor structure of the questionnaire against the background of this particularly challenging pandemic situation, a confirmatory factor analysis (CFA) was calculated, which was evaluated by determining the fit indices.

## 3. Results

The data collected in this study indicate a connection between the respective phase of the pandemic, including the dynamic intensity of the associated consequences and the severity of perceived life stress of first-year medical students. Analyses of variance (ANOVAs) were performed to examine the differences between the distinct points in time, always including the different years (20/21, 21/22, 22/23) as between subject factors (Table 3). Avoiding the error of multiple comparisons, Bonferroni–Holm-corrected *p*-values were reported. The 95% confidence intervals were calculated to ensure better insight into the nature of the data structure (Table 3).

**Figure 1:**
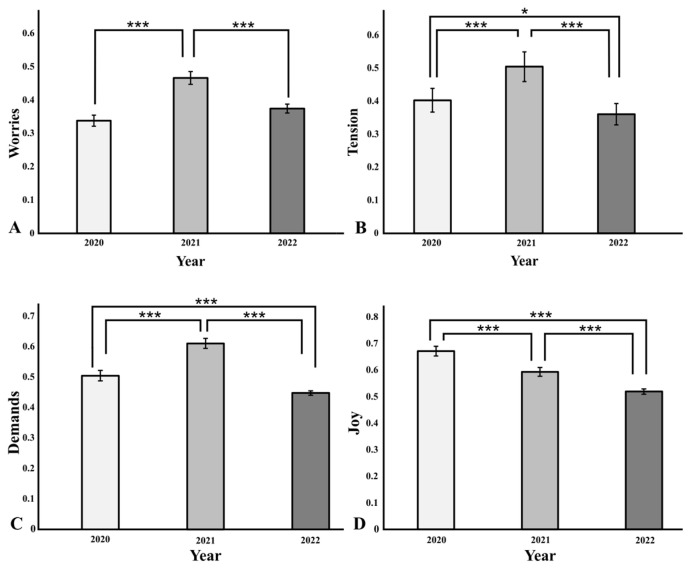
(**A**–**D**) Bar charts of perceived worries, tension, demands and joy among three different cohorts of first-year medical students over the period of three consecutive winter semesters (2020, 2021, 2022). Asterisks indicate: * = *p* < 0.05, *** = *p* < 0.001.

Here, we demonstrate a significant increase in the factors worries, tension and demands, as well as a significant and steady decrease in the construct of general joy of life in the winter semester 2021/2022, at the peak of the COVID-19-associated restrictions (Figure 1).

The examined factor worries differed significantly in the comparison of the three consecutive winter semesters (F(2, 575) = 11.13, *p* < 0.001, partial η^2^ = 0.037). At the beginning of the winter semester 21/22, the factor of worries was significantly higher than 20/21 (*p* < 0.001) and 22/23 (*p* < 0.001), whereas no significant difference between the years 20/21 and 22/23 (*p* = 0.08) could be observed (Figure 1).

A significant difference for the factor of generally perceived tension was also found (F(2, 575) = 21.55, *p* < 0.001, partial η^2^ = 0.070). The perceived tension at the beginning of the winter semester 21/22 was significantly higher than 20/21 (*p* < 0.001) and 22/23 (*p* < 0.001). Equally, the values for perceived tension in the winter semester 20/21 were significantly higher than at the beginning of the winter semester 22/23, albeit with a smaller effect size (*p* = 0.048) (Figure 1).

Similarly, significant differences were shown for the factor demands (F(2, 575) = 45.32, *p* < 0.001, partial η^2^ = 0.136). At the beginning of the winter semester 21/22, the factor of demands was significantly higher than 20/21 (*p* < 0.001) and 22/23 (*p* < 0.001). It could also be demonstrated that the perceived demands in 20/21 were rated as significantly higher than in the winter semester 22/23 (*p* < 0.001) (Figure 1).

With regard to the factor joy, significant differences could also be identified over the course of the three consecutive years (F(2, 575) = 31.52, *p* < 0.001, partial η^2^ = 0.099). While the factor perceived joy was most pronounced in the winter semester 20/21, it was already significantly reduced in the following year 21/22 (*p* < 0.001) and continued to decrease towards the winter semester 22/23 (*p* < 0.001) (Figure 1).

To verify the questionnaire’s factor structure regarding the addressed target group of German medical students during this particularly challenging period and to investigate the correlations between the latent factors, a Confirmatory Factor Analysis was performed. The corresponding factor loadings were calculated and further analyzed (Table 4).

**Figure 2:**
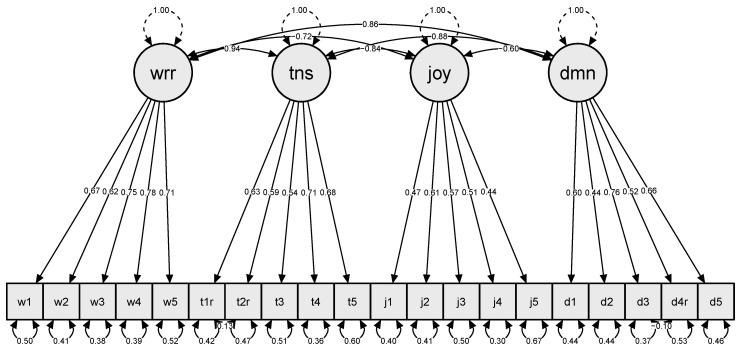
Confirmatory Factor Analysis. Abbreviations indicate: wrr: worries, tns: tension, joy = joy, dmn = demands.

The fit indices CFI (0.908), RMSEA (0.071) and SRMR (0.052) indicated a quite acceptable model fit, although the chi-square test was significant (*p* < 0.001). Strong positive correlations could be found between worries and tension (0.94), worries and demands (0.86), and tension and demands (0.876), whereas strong negative correlations could be observed between worries and joy (−0.72), tension and joy (−0.84), and joy and demands (−0.60) (Figure 2).

**Figure 3:**
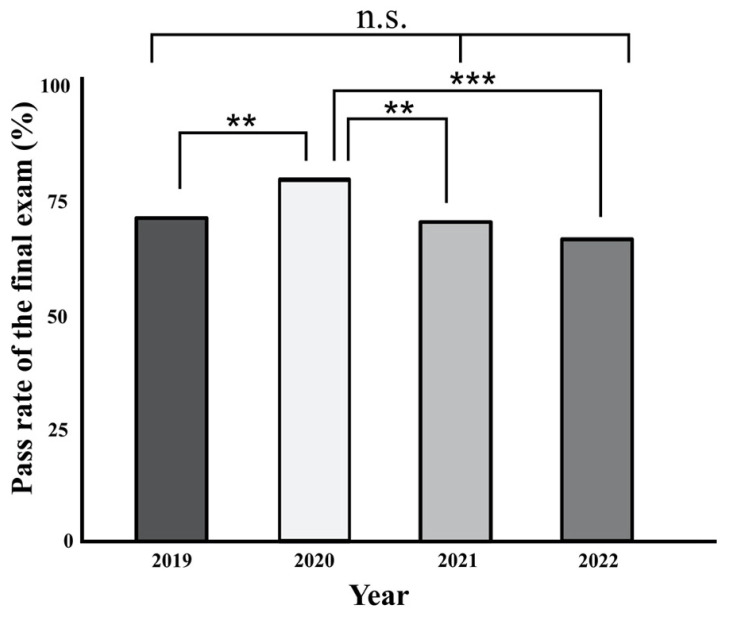
The figure indicates the pass rate of the Anatomy I exam of the first semester in medical studies over the years 2019, 2020, 2021 and 2022. Asterisks indicate: ** = *p* < 0.01, *** = *p* < 0.001. n.s.: not significant.

The analysis of academic performance with regard to the reference year 2019 and the years of the ongoing pandemic 2020, 2021, 2022 revealed that students in 2020 had the highest pass rate. Except for the pass rate in 2020, the respective students have otherwise shown a slight downward trend in terms of performance since 2019, which, however, is not significant (Figure 3). The pass rate of the Anatomy I exam differed significantly when comparing the years 2019, 2020, 2021 and 2022 (F(3, 1533) = 8.31, *p* < 0.001, partial η^2^ = 0.016). The pass rate in 2020 was significantly higher than in the previous year 2019 (*p* = 0.009) and significantly higher than in the following years 2021 (*p* = 0.002) and 2022 (*p* < 0.001). In 2019, the pass rate was slightly higher than in 2021 (*p* = 0.651) and 2022 (*p* = 0.366), but not significantly. In 2022, the pass rate was slightly reduced compared to 2021 (*p* = 0.522).

## 4. Discussion

This study points to the notion that there is a clear connection between the course of the COVID-19 pandemic and the associated variable intensity of the effects on social and university life and the expression of students’ perceived life stress. A comprehensive corpus of scientific papers has already examined the effect of the COVID-19 pandemic on perceived stress levels of student populations across various disciplines [51,52,53,54]. While an analysis of 13 studies regarding the impact of the COVID-19 pandemic on the mental health status of medical students already pointed to increased levels of anxiety and stress [55], this study was able to identify the dynamics of perceived stress among first-year medical students caused in association with the ongoing pandemic.

Not only because of the described effects of experienced stress on cognitive processes and motivational factors [41,42,51,56,57], it is a relevant construct for the entire education sector. Additionally, it has already been empirically proven that academic stress is a relevant risk factor for mental health problems [52,53,54,56], which in turn can cause lower academic functioning and is considered a predictor of dropout among students in higher education [58,59,60,61].

Interestingly, in comparison to the obtained factors worries, tension and demands, the factor joy—which relates to general perceived joy of life—showed different dynamics in its expression over the given period of three years. In contrast to the factors worries, tension and demands, which increased at the peak of the COVID-19-related restrictions and then fell again, the factor joy recorded a continuous, significant decrease in the observed period of 3 years. This finding can also be discussed against the background of a more global data situation. Global survey data of more than 150 countries, analyzed and published by the Sustainable Development Solutions Network, revealed that in 2020, negative affect, as indicated by worry and sadness, increased by 8% compared to the preceding pre-pandemic years [62]. Against the backdrop of this globally collected data, this negative affect metric then dropped to 3% above baseline in the following year, which can be interpreted in terms of emerging resilience or habituation [62]. However, the data generated in this study, which show a continuous decline in general joy of life among first-year medical students, therefore underline the necessity of identifying local influencing factors for the evaluation of mental stress parameters in the respective environment. As a first interpretive approach regarding the lack of an increase in joy of life after the peak of the COVID-19-associated restrictions in the winter semester 21/22, the Russian invasion of Ukraine (24 February 2022) should be considered, since the serious consequences of this war of aggression caused and still cause great fear and uncertainty in the surrounding countries, including Germany [63]. The trend shown here regarding the steady decline in general joy of life among first-year medical students should be considered as an alarming signal, since joy of life is known to be a protective factor, strengthening resilience against mental illnesses [64,65]. This becomes particularly relevant in situations such as the COVID-19 pandemic, as designated protective factors such as perceived social support appear to be diminished [66,67,68].

Additionally, the data described here indicate that dynamics in academic performance during the pandemic may be difficult data to correlate, as the implications of COVID-19-associated changes in education also include alterations in assessment strategies. The sudden shift to online assessment is often discussed against the backdrop of safeguarding academic integrity due to the often-inadequate supervision of higher education exams [69,70,71]. In the data shown here, the Anatomy I exam was taken online only in 2020, which resulted in a significantly higher pass rate and should be discussed in light of the different conditions compared to the reference year 2019 and the subsequent years 2021 and 2022. Excluding the year 2020, however, a slight decrease in academic performance is evident, but this cannot be considered statistically significant. The fact that increased subjectively perceived stress does not necessarily have to be reflected in a sharp drop in academic performance is embedded in the context of the previous literature [16,17], which, conversely, cannot imply that a stable academic performance can indicate satisfactory external factors such as perceived stress.

In addition to the significance and social relevance of the data described, reference should be made to certain limitations contained in this study design. While the PSQ is a sufficiently validated and tested survey instrument for perceived life stress [47,49,72,73], here it could be shown that some factor loadings drop below 0.7; the factors tension and demands especially load quite inhomogeneously. These obtained findings encourage a discussion of the extent to which exceptional demanding situations such as the COVID-19 pandemic influence the factor structure of the PSQ.

Furthermore, although the data show a strong increase in the factors worries, tension and demands, no conclusions can be drawn about the individual attribution of certain stressors. This concern of individual data collection likewise limits the direct predictive power of subjectively perceived stress on academic performance, as this study focuses on the dynamic changes in the mean scores of the associated constructs. Follow-up studies should therefore break down specific subpopulations within vulnerable groups and follow individual parameter expressions to provide more holistic information on the impact of crisis situations on mental stress parameters along with academic achievement. For a more integrated comprehension, more objective, physiological markers of chronic stress could also be collected, such as immune markers, circulating glucocorticoids or catecholamines. Since the sample size in 2020 and 2021 is lower than in 2022, the potential for response bias as a possible source of error in standardized questionnaire-based surveys should be discussed. However, it has to be emphasized that at the beginning of each winter term, the medical students were randomly assigned to small groups by the Dean’s Office of the Medical Faculty. Since, for infection prevention reasons, only a reduced number of randomly selected small groups were able to attend the seminars in presence in 2020 and 2021, correspondingly less data were generated within the survey period. While it would have been desirable to examine three similarly sized sample numbers, it should be noted that the acquisition of the three data sets was conducted under the same randomization and conditions.

The issue of perceived stress in young college entrants is a widely discussed and studied area in the scientific cosmos, reflecting the relevance of studying associated stressors and further predictors of general well-being. Our present results should contribute to this by illustrating the variable intensity of the influence of external stressors, such as the ongoing COVID-19 pandemic.

## 5. Conclusions

This repeated cross-sectional study was able to demonstrate that the factors worries, tension and demands of first-year medical students were significantly increased at the peak of the COVID-19-associated restrictions in the winter semester 21/22 compared to the previous (20/21) and the following (22/23) winter semesters. In addition, a continuous decline in general joy of life could be identified. Here, we describe the emerging dynamics of the influence of the COVID-19 pandemic on stress experienced by first-year medical students—a particularly vulnerable group regarding mental health parameters. Health hazards, political decisions and how the community deals with crisis situations are directly related to perception, behavior and ultimately to elementary mechanisms of a functioning society. These results should also be used to create awareness among the faculties in order to develop protective measures that take into account the influence of dynamic exogenous stressors on university life.

## Figures and Tables

**Table 1:** Demographic characteristics.

	2020	2021	2022	Total
Number of total participants	126	116	336	578
Male, *n* (%)	34 (26.98)	36 (31.03)	107 (31.85)	177 (30.62)
Female, *n* (%)	92 (73.02)	80 (68.97)	227 (67.56)	399 (69.03)
Diverse *, *n* (%)	-	-	2 (0.60)	2 (0.35)
Age, mean (SD)	20.03 (2.60)	22.00 (3.18)	20.40 (3.10)	20.63 (3.09)

Note. SD = Standard Deviation, * here, the term diverse is used to refer to persons who do not conform to socially defined male or female gender norms.

**Table 2:** Factor description 2020–2022.

Factors	Items	2020				2021				2022			
	(*n*)												
		Rating	Mean	SD	Skewness	Rating	Mean	SD	Skewness	Rating	Mean	SD	Skewness
Worries	5	3.33	0.66	0.20	0.18	4.67	0.93	0.21	0.49	3.74	0.75	0.24	0.47
Tension	5	4.03	0.81	0.18	0.51	5.05	1.01	0.17	0.05	3.61	0.72	0.22	0.28
Joy	5	6.75	1.34	0.20	−1.38	5.93	1.19	0.18	−0.09	5.20	1.04	0.18	−0.04
Demands	5	5.05	1.01	0.19	0.15	6.11	1.22	0.18	−0.37	4.48	0.90	0.14	−0.18

Note. *n* = number of items per construct, Rating = overall factor ratings, Mean = mean value of each item of the factor, SD = Standard Deviation.

**Table 3:** Analysis of variance.

Factors	ANOVA				Post Hoc Analyses		
					2020 × 2021	2020 × 2022	2021 × 2022
	df	F	*p*	η^2^	*p* [95% Cl—Lower and Upper Bound]		
Worries	575	11.13	<0.001	0.037	<0.001 [0.065, 0.203]	0.081 [−0.014, 0.097]	<0.001 [−0.150, −0.035]
Tension	575	21.55	<0.001	0.070	<0.001 [0.040, 0.163]	0.048 [−0.092, 0.001]	<0.001 [−0.195, −0.092]
Demands	575	45.32	<0.001	0.136	<0.001 [0.057, 0.154]	<0.001 [−0.096, −0.018]	<0.001 [−0.203, −0.123]
Joy	575	31.52	<0.001	0.099	<0.001 [−0.134, −0.021]	<0.001 [−0.198, −0.106]	<0.001 [−0.121, −0.026]

Note. ANOVA = Analysis of variance, df = degrees of freedom, F = F-value, *p* = *p*-value, η^2^ = partial eta-squared, CI = Confidence intervals.

**Table 4:** Factor loadings.

Indicator	Estimate	*p*	95% Confidence Interval	
			Lower	Upper
w1	0.674	<0.001	0.601	0.746
w2	0.624	<0.001	0.558	0.690
w3	0.755	<0.001	0.686	0.824
w4	0.777	<0.001	0.707	0.848
w5	0.714	<0.001	0.639	0.788
t1r	0.632	<0.001	0.565	0.699
t2r	0.589	<0.001	0.521	0.657
t3	0.544	<0.001	0.475	0.613
t4	0.705	<0.001	0.639	0.772
t5	0.684	<0.001	0.607	0.761
j1	0.466	<0.001	0.402	0.531
j2	0.613	<0.001	0.544	0.682
j3	0.571	<0.001	0.499	0.644
j4	0.513	<0.001	0.454	0.572
j5	0.441	<0.001	0.362	0.519
d1	0.598	<0.001	0.530	0.666
d2	0.435	<0.001	0.372	0.498
d3	0.758	<0.001	0.686	0.829
d4r	0.523	<0.001	0.451	0.596
d5	0.659	<0.001	0.588	0.730

Note. *p* = *p*-value, w = worries, t = tension, j = joy, d = demands, r = reverse coded item.
